# Brazilian Adolescents Infected by HIV: Epidemiologic Characteristics and Adherence to Treatment

**DOI:** 10.1100/tsw.2009.136

**Published:** 2009-11-18

**Authors:** Julia K.C. Machado, Maria J. C. SantʼAnna, Veronica Coates, Flavia J. Almeida, Eitan N. Berezin, Hatim A. Omar

**Affiliations:** ^1^ Department of Pediatrics and Adolescent Medicine, Santa Casa de Sao Paulo School of Medicine, Sao Paulo, Brazil; ^2^ Department of Pediatrics and Infectious Diseases, Santa Casa de Sao Paulo School of Medicine, Sao Paulo, Brazil; ^3^ Department of Pediatrics and Obstetrics/Gynecology, University of Kentucky, Lexington, KY, USA

**Keywords:** adolescents, HIV, adherence, epidemiology

## Abstract

Over the last 3 decades since the first AIDS cases appeared, we have witnessed great progress in therapeutic methodologies that have transformed the evolution of the disease from debilitating and fatal, into chronic and controllable. HIV-infected children are arriving at adolescence and bringing specific challenges, not only to themselves, but also to their families and caregivers. This retrospective study sets forth epidemiological and treatment characteristics of 46 HIV-infected adolescents followed in a specialized university service relating said characteristics to therapy adherence assessed through a combination of three indirect methods. Therapy adherence did not reveal any association with either epidemiologic characteristics regarding age, sex, school level, household composition, age at diagnosis, mode of infection, knowledge of diagnosis, treatment time, or initial antiretroviral scheme. Patients with good therapy adherence presented lower viral load and used a smaller number of antiretroviral schemes.

## INTRODUCTION

Adolescence, defined by the World Health Organization (WHO) as the period from 10 to 19 years of age[1], presents peculiar behavioral characteristics. This is the time of discovering one's own identity, the unfolding development of opposition between parents and teenagers, the questioning of values learned in childhood, and humor fluctuations. This phase gives rise to the need to envision an imaginary future and depart from the present, as well as to a sensation of invulnerability and all-powerfulness. Sexuality is developed and there is the need to belong to a group[2]. All these facts together generate various conflicts in connection with the chronic and stigmatizing disease of acquired immunodeficiency syndrome (AIDS), which requires a definite personal commitment for successful therapy evolution.

Going through adolescence with human immunodeficiency virus (HIV) brings specific challenges, not only to youngsters, but also to their families and caregivers[3,4]. It is necessary to reflect on therapy adherence, posology, diagnosis disclosure, and life planning, in addition to the adolescent's decision making concerning treatment, relationships, intimacy, school, and professional career[5,6].

There are approximately 33 million people infected with HIV worldwide. The global percentage of infected adults is stable since 2000; however, the absolute number of HIV-seropositive persons is increasing. The reason for this is that people on HIV therapy live longer and the number of newly infected individuals exceeds the number of deaths[7]. Since the 1980s until June 2008, the Brazilian Federal Health Department (MS) recognized 506,499 AIDS cases in Brazil, including 11,079 in young people between 13 and 19 years of age[8].

Over the last 3 decades since the first AIDS cases appeared, we have witnessed great progress in therapeutic methodologies that have transformed the evolution of the disease from debilitating and fatal, into chronic and controllable[9,10]. Highly active antiretroviral therapy (HAART) is a combination of antiretroviral medications that prevent HIV replication in different stages of its cycle, and has demonstrated the capacity for increasing life expectancy and quality of life, in addition to decreasing transmission risk[11]. In order to meet these objectives, high levels of therapy adherence are required, i.e., no less than 95%[12,13,14]. In this context, therapy adherence may be defined as the proportion of health-related behaviors that comply with applicable medical recommendations[13]. Whenever the foregoing condition is not met, medication serum levels remain at suboptimal levels, allowing HIV multiplication. On the other hand, the latter situation may set off resistance, not only to available medications and cause loss of effectiveness, but also to other medications that could feature a cross-resistance pattern and at times to entire drug categories[15].

Young people between 15 and 24 years of age represent approximately 45% of newly HIV-infected persons worldwide[7]. Recent studies made in the U.S. suggest that most adolescents and young adults from 13 to 24 years of age are infected through sex: males through homosexual contact, with a small percentage through injection drug use or heterosexual contact; females through heterosexual contact, with a very small percentage through injection drug use[16]. Brazilian data up to 2005 showed that among young males from 10 to 19 years of age, 33% of HIV transmission was attributed to injection drug use, 21% to homosexual contact, and 14% to heterosexual contact. For the same age bracket, young female transmission through heterosexual contact represented 70% and injection drug use amounted to 13% of new infections[17].

On a worldwide basis, 370,000 children and adolescents under 15 years of age were infected with HIV in 2007, which increased the number of HIV-seropositive children and adolescents from approximately 1.6 million in 2001 to nearly 2.0 million in 2007. Estimates indicate that an excess of 90% of infected children acquired HIV infection through vertical transmission (mother-to-child transmission at delivery, during breastfeeding, or pregnancy). A small fraction of HIV transmission to children was through transfusion of blood and blood products, sexual abuse, contaminated syringes, or sexual activity (the latter being important among adolescents)[7].

In the absence of therapy, AIDS advances rapidly during the first year of life in approximately 20% of HIV-infected children[18]. The introduction of HAART made possible an important reduction of morbidity and mortality in children[19,20], as therapy failure is frequently related to inappropriate therapy adherence[21,22]. With therapy, many children are able to reach adolescence; some with poor clinical evolution, however, but most of them with improved life expectancy[23,24].

As a result of the advance in pediatric knowledge, children with chronic conditions now have a greater life expectancy. This has, however, led to an increase in the number of adolescents with chronic diseases[25,26]. These young people face a greater probability of undergoing psychosocial isolation and having low self-esteem, and are frequently associated with risk behaviors, such as dropping out of school, drug addiction, and unprotected sexual activity[27,28,29]. Difficulty in treatment adherence is common among adolescents with chronic diseases and even more so among those who use antiretroviral therapy (ART)[30].

AIDS is frequently associated with cognitive impairment, parents' illness and death, breakdown in family structures, depression, and behavioral disturbances[31,32]. Moreover, patients may also require complex therapy schemes due to their experience in previous treatments[33]. ART toxicity and complexity constitute important challenges in patients' life contexts[34,35]. On the other hand, we know that a favorable response to HAART depends on virus sensitivity to medications and quasiperfect adherence[33,36]. Reduction in viral load (HIV-1 RNA copies in the circulation) is the fastest parameter for assessment of treatment response[37,38].

Direct and indirect methods for adherence assessment are described herein, consistent with measurement type. Each method has advantages and disadvantages, yet none of them offers an accurate evaluation. Direct or objective methods are more expensive and difficult to apply, such as electronic monitoring devices and measurement of serologic levels of medications, among others. Indirect or subjective methods include evaluation of laboratory parameters on therapy response through viral load and CD4 percentage, self-reports, pill counting, and adherence assessment by health professionals. In the absence of a gold standard, it is assumed that a combination of diverse methods is the best way to evaluate adherence[13].

## OBJECTIVES

- Describe epidemiologic data about HIV-infected adolescents: age, gender, Tanner stage of sexual development, school level, household nucleus composition, age at diagnosis, transmission mode, diagnosis knowledge, and viral load
- Describe ART characteristics of said patients: treatment time, first ART scheme used, and the number of ART schemes used
- Correlate epidemiologic and ART characteristics to therapy adherence

## METHODS

This is a descriptive and retrospective study.

We analyzed 65 medical records of 10- to 19-year-old adolescents diagnosed with HIV prior to the studied period, all of whom were followed-up at the Pediatric Infectology Ambulatory of Santa Casa de Sao Paulo (PIASCSP). We collected data from consultations held between September 14, 2007 and March 28, 2008. Only the medical records of 46 patients who attended medical consultations during the above-mentioned period were included. Those patients were all under antiretroviral treatment. The 19 remaining medical records were excluded for the reason that those patients did not attend consultations during the above-specified period.

Registered data included age, gender, Tanner stage of sexual development, school level, household composition, age at diagnosis, transmission mode, diagnosis knowledge, viral load, ART time, initial antiretroviral scheme used, number of antiretroviral schemes used, and therapy adherence.

School level was categorized as either appropriate or delayed. The concept of delay was in accordance with the applicable National Educational Bases and Guidelines Act specifying that mandatory elementary school should start at 6 years of age and last for 9 years, with three additional years of study for high school completion. The age considered ideal for each school year should have been reached in the period from the second half of the preceding year and the first half of the current year[39].

HIV-1 viral load quantification was accomplished by the Adolpho Lutz Central de Sao Paulo Institute through b-DNA, kit HIV 3.0 RNA tests. The minimum limit of detection was 50 copies per milliliter and the maximum was 500,000 copies per milliliter. By reason of a wide range of viral load measurements, the statistical tests were made by applying log transformation and replacing the undetectable by 49. There were no measurements above the maximum limit of detection.

For the duration of the follow-up held at the PIASCSP, viral load tests were carried out every 3 or 4 months in accordance with applicable Federal Health Department (MS) instructions.

Schemes with three or more concurrent antiretroviral medications were defined as HAART.

The number of used antiretroviral schemes only considered changes caused by therapy failure. Changes required by reason of drug interaction or drug production interruption were not considered as new schemes.

Therapy failure was defined as occurrence of clinical deterioration and/or worsening in immunologic and/or virologic laboratory parameters in accordance with applicable MS criteria[22,37] (Table 1).

**TABLE 1**
**Therapy Failure Criteria[22]**

Clinical criteria

- Neurological deterioration
- Growth failure and/or failure of neuropsychomotor development
- New clinical progress indicator event (except for the context of immune reconstitution)

Immunological criteria

- Change in immunological category
- Reduction >20–25% in absolute CD4 lymphocyte count (in relation to the highest value reached in current scheme) in no less than two serial measurements, in the absence of recurrent infection that would justify a transient reduction in CD4
- For children with levels of CD4 <15%, persistent reduction ≥5% (e.g., from 15 to 10%, or from 10 to 5%)

Virological criteria

- Virological response below minimum acceptable response after 3–6 months of therapy: reduction <0.7–1.0 log, dependent on antiretroviral scheme used in relation to initial level
- Persistent viral load increase after starting of therapy >0.5 log
- Persistent viral load return to detected level subsequent to undetected period

Adherence assessment was carried out by the Pediatric Infectology Team of Santa Casa de Sao Paulo (SCSP) based on a combination of the following three indirect methods:

1. Assessment of laboratory parameters on therapy response: viral load and CD4 count
2. Self-report considering answers of both patient and patient companion to “Adherence Approach” Questionnaire (Table 2)
3. Estimate made by health professionals reflecting overall team member’s perception of patient considering his/her assiduity, interest, and attitude[40]

Therapy adherence was categorized into three levels: good, doubtful, and inadequate.

Descriptive analysis percentages were used for qualitative variables and measurement summaries (average, standard deviation, median, minimum, and maximum), and boxplots for quantitative variables.

In the study on association between variables, we used Kruskal-Wallis and Mann-Whitney tests. These are nonparametric tests that should be used when you are not able to assume that your data area was sampled from Gaussian populations. The Mann-Whitney test is used to study two independent groups and the Kruskal-Wallis when you have more than two groups[41]. Significance level ($\alpha$) considered was 5%.

The analyses were made using Epi Info version 3.4.1 and SPSS version 13.0 software for Windows.

**TABLE 2**
**"Adherence Approach" Questionnaire**

- How many doses did you forget to take in the last 3 days?
- Does the patient companion know the doses and the timetable?
- Is there any difficulty in taking the medication?
- Does anybody see the patient taking the medication?

## RESULTS

Patient ages varied from 10 to 19 years, with both average and median equal to 14 years; 50% of these patients were female and 50% were male.

Tanner stage records were either missing or incomplete in most medical records, which made it impossible to make use of said data.

School level was considered delayed for 50% of patients.

As to household composition, 63% of patients lived with their biological father and/or mother; 30% with other relatives, and 7% with adoptive parents.

The average age at diagnosis was 5 years (minimum age was 1 month and maximum age was 15 years) and the median age at diagnosis was 4 years.

Mode of transmission was predominantly vertical (85%), with 15% unknown contamination.

Eighty-three percent of patients knew their HIV-infection diagnosis. The 17% who did not know their diagnoses were 10–13 years of age (average age was 11), and their families had not authorized disclosure of the diagnosis and had strong resistance to do so.

Median viral load at the time of medical record analysis was 3.17 log (1.69–5.16), the average being 3.09 log.

Average and median treatment times were both equal to 8 years, with minimum equal to 3 months and maximum equal to 15 years.

Thirty-nine percent of the patients started therapy with three antiretroviral drugs (HAART), 41% with two antiretroviral drugs (double drug therapy), and 20% started with only one drug (monotherapy).

The average number of antiretroviral schemes used by each patient was four (3.2 standard deviations), ranging from one to 14, with median value of three. Twenty-two percent of patients used only one antiretroviral scheme, 30% used from two to three, 33% used from four to six, and 15% used seven or more antiretroviral schemes.

Therapy adherence was considered good in 48% of patients, doubtful in 26%, and inadequate in 26%.

Female patients did not present a statistically significant difference in adherence in relation to male patients (Table 3).

No statistically significant adherence difference was detected between patients of appropriate school level and those who were delayed, or between patients who knew their diagnoses and those who did not (Table 3).

No association was detected between adherence and age, household composition, age at diagnosis, transmission mode, treatment time, or with regard to the first antiretroviral scheme used (Tables 3 and 4).

Viral load demonstrated association with therapy adherence, with $p = 0.0001$. There was no significant viral load difference between patients whose adherence was considered doubtful and inadequate (Fig. 1, Table 4).

The number of antiretroviral schemes used was associated with therapy adherence ($p = 0.012$). There was no statistically significant difference in the number of antiretroviral schemes used between patients of doubtful or inadequate therapy adherence (Fig. 2, Table 4).

**TABLE 3**
**Characteristics of Patients in Each Therapy Adherence Level**

| Characteristics | *n* (%) | Therapy Adherence | | |
|---|---|---|---|---|
| | | Good | Doubtful | Inadequate |
| Gender | 46 (100%) | | | |
| Male | 23 (50%) | 11 (48%) | 5 (22%) | 7(30%) |
| Female | 23 (50%) | 11 (48%) | 7 (30%) | 5 (22%) |
| School level | 46 (100%) | | | |
| Appropriate | 23 (50%) | 12 (52%) | 7 (31%) | 4 (17%) |
| Delayed | 23 (50%) | 10 (43%) | 5 (22%) | 8 (35%) |
| Household composition | 46 (100%) | | | |
| Biological parents | 29 (63%) | 17 (59%) | 8 (27%) | 4 (14%) |
| Other relatives | 14 (30.5%) | 5 (36%) | 4 (28%) | 5 (36%) |
| Adoptive parents | 3 (6.5%) | 0 | 0 | 3(100%) |
| Transmission mode | 46 (100%) | | | |
| Vertical | 39 (84.8%) | 17 (43%) | 10 (26%) | 12(31%) |
| Unknown | 7 (15.2%) | 5 (71%) | 2 (29%) | 0 |
| Diagnosis knowledge | 46 (100%) | | | |
| Yes | 38 (82.6%) | 17 (45%) | 10 (26%) | 11(29%) |
| No | 8 (17.4%) | 5 (63%) | 2 (25%) | 1 (12%) |
| First ART scheme used | 46 (100%) | | | |
| Monotherapy | 9 (19.6%) | 5 (56%) | 2 (22%) | 2 (22%) |
| Double drug therapy | 19 (41.3%) | 4 (21%) | 6 (32%) | 9 (47%) |
| HAART | 18 (39.1%) | 13 (72%) | 4 (22%) | 1 (6%) |

## DISCUSSION

We are in the third decade of the AIDS pandemic. The progress achieved in HIV-infection treatment transformed the prognosis and life expectancy of HIV-seropositive people. HIV-infected children reach adolescence and face the challenge of conciliating a therapy that does not admit failings, and the process of discovery and development of one's own identity surrounded by stigma, prejudice, and social discrimination. An atmosphere of secrecy makes it difficult to take fundamental development steps as to belong to a group or exercise sexuality[42].

It is typical that adolescents have the so-called "magic thought" that gives them a sense of invulnerability. Therefore, we have HIV-seropositive adolescents who believe they will not be harmed by the disease and consequently abandon their treatment until the immunodeficiency develops in an incontestable manner, forcing them to return to health care. The number of adolescents registered in PIASCSP when this research was made was 65, however, only 46 (70.8%) were under regular follow-up, i.e., attended monthly or bimonthly consultations.

Other typical characteristics of adolescents also compromise therapy adherence. Their unstable personalities may cause them to either comply with or challenge medical recommendations. There is a contradiction between roles, sometimes of a dependent child, sometimes of a self-sufficient individual. It is common for adolescents to fantasize an imaginary future and depart from the present, as if passively viewing his/her own life as it happens. In addition, there is a distorted perception of time that gives rise to health care postponement on account of irrational urgencies.

**TABLE 4**
**Measurement Summary of Age, Age at Diagnosis, Viral Load (log), Treatment Time, and Number of Antiretroviral Schemes Used in Each Therapy Adherence Level**

| Measurement Summary | Therapy Adherence | | | *p* |
|---|---|---|---|---|
| | Good | Doubtful | Inadequate | |
| Age (years) | | | | 0.7425 |
| Number of patients | 22 | 12 | 12 | |
| Average | 14.05 | 14.97 | 13.83 | |
| Standard deviation | 2.45 | 3.39 | 2.39 | |
| Median | 13.70 | 14.03 | 13.68 | |
| Age at diagnosis (years) | | | | 0.4461 |
| Number of patients | 22 | 12 | 12 | |
| Average | 6.23 | 4.67 | 4.74 | |
| Standard deviation | 4.26 | 4.91 | 3.33 | |
| Median | 4.46 | 2.67 | 4.67 | |
| Viral load (log) | | | | 0.0001* |
| Number of patients | 22 | 12 | 12 | |
| Average | 2.26 | 3.59 | 4.11 | |
| Standard deviation | 0.94 | 1.07 | 0.97 | |
| Median | 1.69 | 4.02 | 4.37 | |
| Treatment time (years) | | | | 0.4471 |
| Number of patients | 22 | 12 | 12 | |
| Average | 7.14 | 9.33 | 8.95 | |
| Standard deviation | 4.74 | 3.15 | 3.38 | |
| Median | 7.28 | 9.34 | 9.23 | |
| Number of ART schemes used | | | | 0.0124* |
| Number of patients | 22 | 12 | 12 | |
| Average | 2.91 | 4.08 | 5.83 | |
| Standard deviation | 3.08 | 1.88 | 3.86 | |
| Median | 1.00 | 4.50 | 4.00 | |

* $p < 0.05$ = statistically significant.

In our study, age did not present any relationship with therapy adherence. The European multicentric study "COHERE" dated 2008 presented an inadequate virological response among children[43]. Mellins et al.[34] and Williams et al.[31] identified a relationship between age increase and inadequate adherence.

In our research, adherence did not demonstrate a difference between male and female patients, while Williams et al.[31] associated female patients to nonadherence. In a study on adults, Nicastri et al.[44] did not find adherence difference among male and female patients, while Collazos et al.[45] identified a better therapy response among female patients.

In our study, school level did not demonstrate association with therapy adherence, although Williams et al.[31] could identify an inadequate school level relationship with nonadherence to treatment. Our result may have been influenced by the Brazilian public education policy that does not allow student failure except in cases of school nonattendance. Furthermore, school performance and interest are also connected with adolescents' plans for life, a relevant theme when dealing with an incurable disease. We could perceive that some families had an attitude of disbelief with regard to the young patient's future in view of his/her disease.

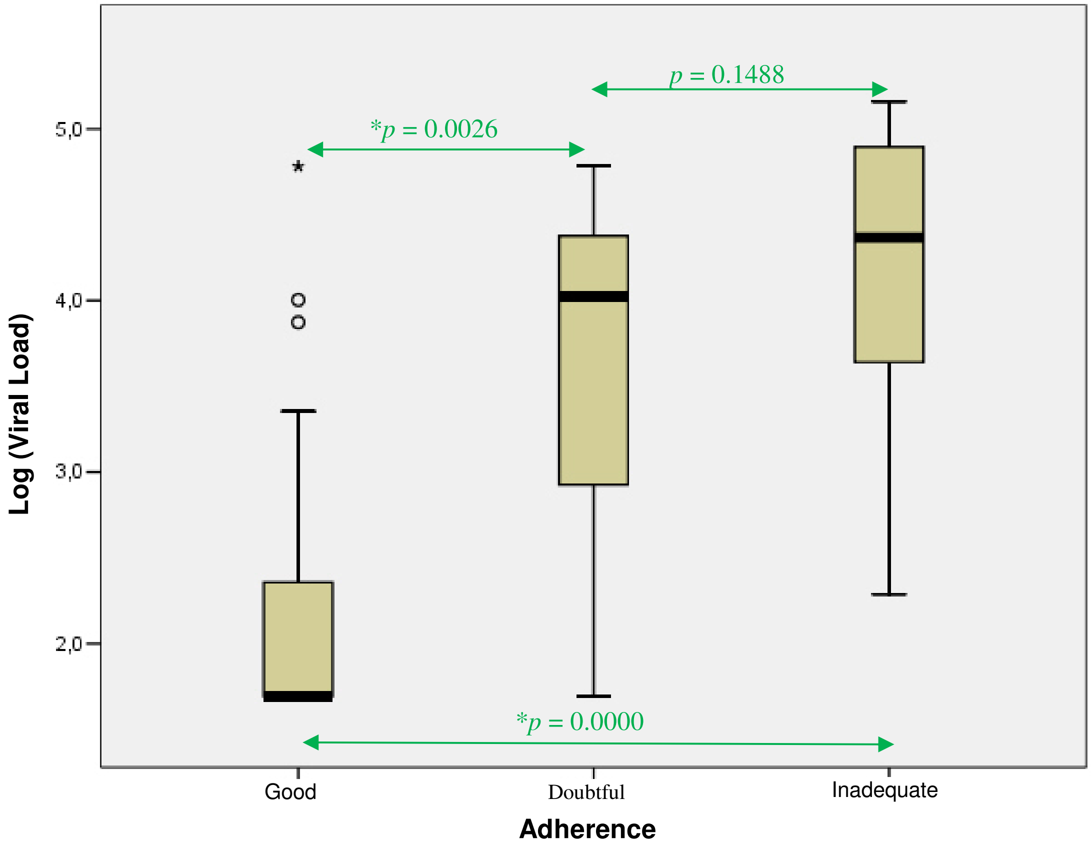

**FIGURE 1.** Boxplot showing viral load distribution consistent with therapy adherence. *$p < 0.05$ = statistically significant.

Our data did not identify any therapy adherence difference in connection with family nucleus, however, only 7% of patients lived with adoptive parents, which may have made said comparison more difficult. In Sao Paulo city, there is a reference hospital for patients who live in institutions. Therefore, our service does not attend said patients, which explains the large percentage of adolescents living with their parents (63%) or relatives (30%). Williams et al.[31], Giacomet et al.[46], and Marhefka et al.[47] associated the presence of adoptive parents or nonrelative caregivers to higher adherence to treatment.

The family context of young people with HIV infection acquired from mother-to-child transmission deserves special attention. Familiarity with other HIV-seropositives in the household and the way they deal with the issue greatly influences adolescent behaviors. We detected higher therapy adherence among children of mothers who were also therapy adherent. On the other hand, in cases where a mother abandons her own treatment and does not take her own medications in the correct manner or frequently requires hospital internment, we detected similar attitudes of her son/daughter. The death of a parent also has a negative impact on young patients, giving them a sensation of powerlessness and discouragement in relation to disease[48].

Although this is a study on adolescents, most of these individuals acquired HIV in childhood. The average age at diagnosis was 5 years with median of 4 years, thus reflecting failure of prenatal diagnosis with regard to the studied sample. The majority of our patients (85%) acquired HIV infection through vertical transmission, which is in harmony with the literature, taking age at diagnosis into consideration. In Brazil, the predominance of vertical transmission reached 84.5% among children under 13 years of age until June 2008[8]. Patients aged 13 and older were 2.4% of vertical transmission cases notified in 2008 in Brazil[8]. This study selected adolescents who were already followed at the PIASCSP, where maximum age

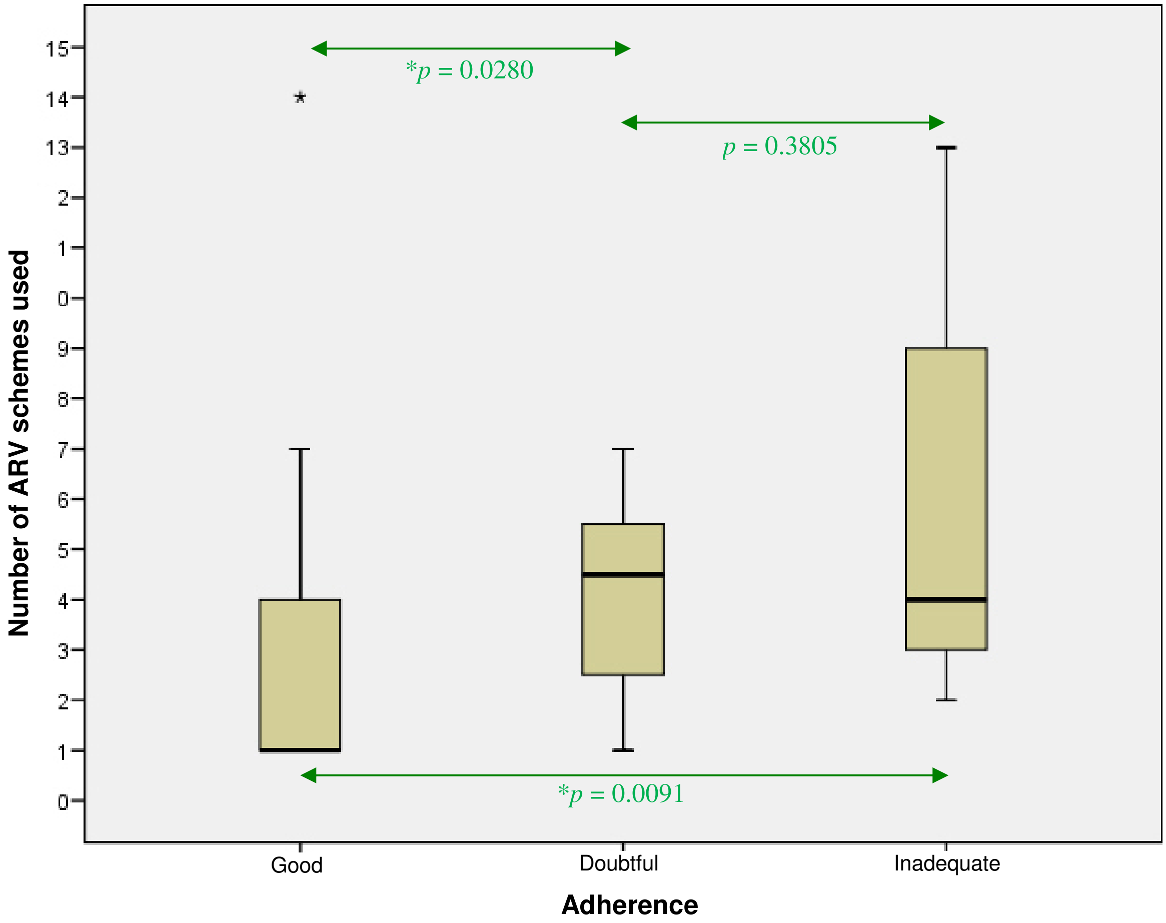

**FIGURE 2.** Boxplot showing number of antiretroviral schemes distribution consistent with therapy adherence. *$p < 0.05$ = statistically significant.

to start follow-up is 13. We believe this fact also contributed to the high rate of vertical transmission we found.

Of the seven patients of unknown contamination, two had received a blood transfusion and two had a history of hospitalization in the 1990s. The other three mentioned either an HIV-infected father or uncle, and one of the patients presented with a perianal condyloma. Considering that sexual abuse by a close relative is frequently referred to in the literature, this hypothesis was indeed considered. However, abuse was never confirmed by either anamneses or physical examination. Not in any of these three cases was it possible to investigate the infected relative as the patient no longer had any communication or contact with that relative.

Our work did not identify any association of therapy adherence with knowledge of diagnosis, which may have been influenced by the large number of patients (83%) who knew about their HIV-seropositive condition. The literature is controversial in relating knowledge of diagnosis to therapy adherence. Williams et al.[31] also did not find any association between therapy adherence and knowledge of diagnosis. Butler et al.[49] did not identify any significant difference in quality of life before or after disclosure of diagnosis. Marques et al.[50] and Battles et al.[48] detected positive repercussions of diagnosis disclosure on therapy adherence. On the other hand, Giacomet et al.[46] studied children (with median age = 8 years) and identified poorer therapy adherence among those patients who knew their diagnoses. In contrast, Biadgilign et al.[51] and Marhefka et al.[47] detected better therapy adherence among patients who did not know their diagnoses.

Some HIV-infected patients became orphans at a very early age and were raised by relatives: aunts, uncles, and grandparents, in particular. These are families that inherited numerous difficulties, such as taking care of children who are incurable and marginalized in society, in addition to having to explain to children what this means. Families often criticize parents in front of their children, blaming them for disease and death. Some families hide the diagnosis from children and even invent a different pathology in order to justify the number of consultations, medical examinations, and medications. Several reasons could explain this attitude, including guilt and shame. Young patients sometimes learn about their disease by themselves, by reading labels in antiretroviral medications and hearing parts of conversations; they initially presume and subsequently discover their condition of being HIV-seropositive. Abadia-Barrero and Larusso[52] identified the worst reactions among adolescents who discovered their diagnoses by themselves. Disease disclosure to a child or adolescent is a delicate and important moment that deserves special care by health team members, relatives, and caregivers.

Adherence assessment carried out by the Pediatric Infectology Team revealed significant association with viral load, a well-documented fact in other studies[14,31,53,54,55].

Our study could not identify any association between treatment time and therapy adherence. Marhefka et al.[47] associated good therapy adherence to lesser duration of HAART.

Therapy adherence comparison among patients who started treatment with different antiretroviral schemes (monotherapy, double therapy, or HAART) did not demonstrate statistically significant differences. Giacomet et al.[46] associated better adherence with HAART use. Murphy et al.[14] demonstrated that survival of HIV-infected adolescents was significantly higher with HAART.

Patients in our study had HIV diagnosis in different years, from 1992 to 2007. That explains the heterogeneity found in the first antiretroviral scheme applied as each patient used whatever antiretroviral was available when they started their therapy. Some of them started treatment at a time when only monotherapy was available, while others started treatment at double therapy phase and more recently, HAART.

We detected a smaller number of antiretroviral schemes used among patients with good therapy adherence (Fig. 2), which is similar to the finding of Souza et al.[56], thus reinforcing the significance of adherence in therapy failure prevention.

Adherence assessed by means of indirect method in our study demonstrated association with viral load and number of antiretroviral schemes used, but not with epidemiologic characteristics of adolescents.

Excellent adherence is fundamental in order to achieve the morbimortality reduction offered by HAART. That could be made difficult by psychosocial questions that emerge intensely during adolescence, in addition to the challenges presented by HIV/AIDS itself. In a country such as Brazil that provides universal access to antiretroviral medications, it is necessary to establish public policies that focus on the overall health of young people and their families or caregivers, with a multidisciplinary approach capable of recognizing and managing the specificities of HIV/AIDS at this stage of life[57,58].

## CONCLUSIONS

Therapy adherence did not present any association with either epidemiologic characteristics of age, sex, school level, household composition, age at diagnosis, mode of infection, knowledge of diagnosis, treatment time, or the initial antiretroviral scheme.

Patients with good therapy adherence presented lower viral load and used smaller numbers of antiretroviral schemes.
